# Transient Exposure to Ethanol during Zebrafish Embryogenesis Results in Defects in Neuronal Differentiation: An Alternative Model System to Study FASD

**DOI:** 10.1371/journal.pone.0112851

**Published:** 2014-11-10

**Authors:** Xavier Joya, Oscar Garcia-Algar, Oriol Vall, Cristina Pujades

**Affiliations:** 1 Unitat de Recerca Infància i Entorn (URIE), Institut Hospital del Mar d'Investigacions Mèdiques, Barcelona, Spain; 2 Red de Salud Materno-Infantil y del Desarrollo (SAMID), Programa RETICS, Instituto Carlos III, Madrid, Spain; 3 Departament de Pediatria, Ginecologia i Obstetricia i de Medicina Preventiva, Universitat Autònoma de Barcelona (UAB), Bellaterra, Spain; 4 Department of Experimental and Health Sciences, Universitat Pompeu Fabra (UPF), Parc de Recerca Biomedica de Barcelona, Barcelona, Spain; Universitat Pompeu Fabra, Spain

## Abstract

**Background:**

The exposure of the human embryo to ethanol results in a spectrum of disorders involving multiple organ systems, including the impairment of the development of the central nervous system (CNS). In spite of the importance for human health, the molecular basis of prenatal ethanol exposure remains poorly understood, mainly to the difficulty of sample collection. Zebrafish is now emerging as a powerful organism for the modeling and the study of human diseases. In this work, we have assessed the sensitivity of specific subsets of neurons to ethanol exposure during embryogenesis and we have visualized the sensitive embryonic developmental periods for specific neuronal groups by the use of different transgenic zebrafish lines.

**Methodology/Principal Findings:**

In order to evaluate the teratogenic effects of acute ethanol exposure, we exposed zebrafish embryos to ethanol in a given time window and analyzed the effects in neurogenesis, neuronal differentiation and brain patterning. Zebrafish larvae exposed to ethanol displayed small eyes and/or a reduction of the body length, phenotypical features similar to the observed in children with prenatal exposure to ethanol. When neuronal populations were analyzed, we observed a clear reduction in the number of differentiated neurons in the spinal cord upon ethanol exposure. There was a decrease in the population of sensory neurons mainly due to a decrease in cell proliferation and subsequent apoptosis during neuronal differentiation, with no effect in motoneuron specification.

**Conclusion:**

Our investigation highlights that transient exposure to ethanol during early embryonic development affects neuronal differentiation although does not result in defects in early neurogenesis. These results establish the use of zebrafish embryos as an alternative research model to elucidate the molecular mechanism(s) of ethanol-induced developmental toxicity at very early stages of embryonic development.

## Introduction

Fetal Alcohol Syndrome (FAS) is the most widely recognized consequence of prenatal alcohol exposure and is the principal preventable cause of mental retardation [1]. The clinical features of FAS can be broadly divided into: growth retardation, morphological malformations (especially craniofacial defects) and impairment in the development of the Central Nervous System (CNS) [2]–[4]. The craniofacial defects include eye abnormalities such as microphthalmia [5], as well as various defects such as: hearing disorders, including ear malformations [6], and thin upper lip [7]. Individuals with all of these categories of defects are at the most severely affected level of alcohol teratogenicity. The term Fetal Alcohol Spectrum Disorder (FASD) is used to describe the majority of FAS-related phenotypes. For this reason, variables such as dose, duration and timing of exposure to alcohol are crucial to confer a certain degree of vulnerability to ethanol-induced teratogenesis [8], [9].

Some of the important and unresolved questions in this field of research are what exactly is(are) the critical period(s) for ethanol exposure during embryogenesis and which of the molecular components expressed during these periods are ethanol-sensitive. The zebrafish model resolves the staging issues, allowing the study of developmental processes in a non-invasive manner under a specific temporal control [10]–[12]. Due to their transparency, internal processes of both embryos and larvae can be easily visualized microscopically allowing in vivo analysis. In order to directly visualize the effect of ethanol exposure in the developing CNS at the cellular level, transgenic zebrafish lines that express fluorescent protein reporters in specific subsets of neurons or in different neural territories can be used. Furthermore, zebrafish embryos develop very rapidly, they are small in size, and the entire nervous system can be observed during development, making zebrafish a suitable model for embryonic studies.

Studies performed using zebrafish as an animal model to investigate the teratogenic mechanisms of prenatal ethanol exposure revealed that these embryos exhibit abnormalities similar to those observed in children diagnosed with alcohol-related birth defects (ARBD) including: developmental retardation [9], reduction of body length or growth retardation [13], abnormal eye development [5], [14], cognitive defects such as aggressive behavior [15], [16], as well as motor deficiencies [17], [18]. Since this cluster of defects overlaps with human FASD, these findings support the view that zebrafish represents an ideal model to study the ethanol effects during pregnancy. However, we wanted to go a step further and focus on the effects of ethanol in specific neuronal cell populations. In this work, we have assessed the sensitivity of specific subsets of neurons to ethanol exposure during embryogenesis using different transgenic fish lines, and characterized the embryonic developmental periods critical for the development of specific groups of neurons.

## Results and Discussion

### Transient ethanol exposure during zebrafish embryogenesis causes morphological malformations characteristics of FASD

**Figure 1:**
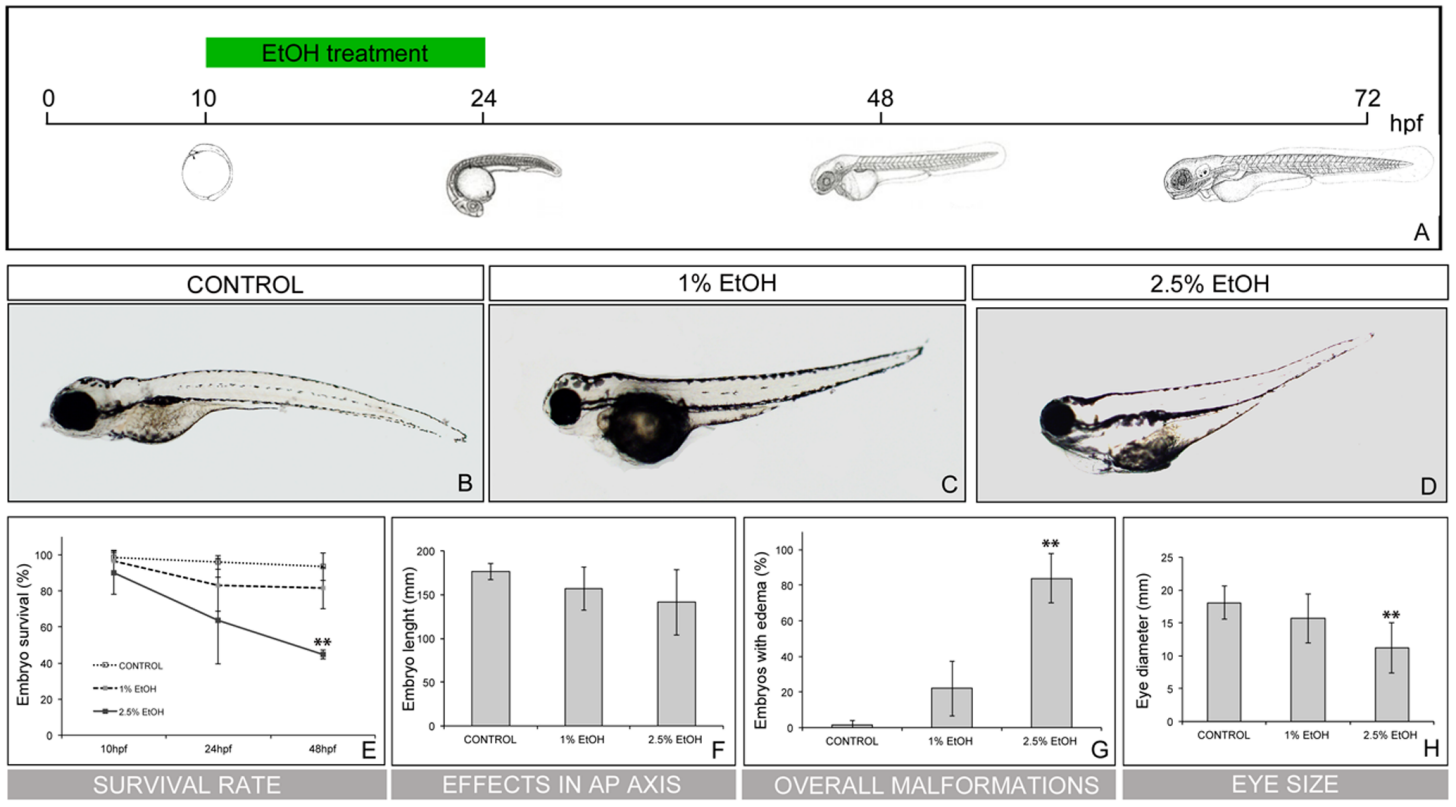
Embryos exposed to ethanol displayed increased incidence of malformations. (A) Scheme depicting the treatment conditions. Zebrafish embryos were initially exposed to 0 (control), 1 and 2.5% ethanol from 10hpf to 24hpf. The embryos were maintained until the desired stages and the phenotypes were analysed. (B-D) Phenotypic analysis of ethanol-treated embryos. Note that treatments above 1% ethanol resulted in visible malformations, namely smaller heads, smaller tails, and underdeveloped eyes. (E) Survival rate at 24 and 48hpf after ethanol-treatment. (F) Effects in embryo body length upon increasing ethanol concentrations. (G) Incidence of ethanol concentration in oedema formation. (H) Eye size analysis. ** p<0.005 vs. control group.

In order to mimic acute ethanol ingestion, we exposed zebrafish embryos during gastrulation and somitogenesis, which are the developmental stages more responsive to ethanol exposure [13], [19], [20]. Bud-stage (10 hours post-fertilization (hpf)) embryos were initially exposed to 0 (control), 1 and 2.5% of ethanol during the next 14 h, similar to previous protocols [20], [21]. Then, ethanol was removed and embryos were maintained without ethanol until the desired development stage, when they were observed (Figure 1A). Embryos transiently exposed to ethanol displayed pericardial oedema, and reduction of body length and eye diameter when compared with control embryos (Figure 1B-D). Interestingly, survival rate (SR) decreased in embryos treated with ethanol, especially in those exposed to higher doses of ethanol (Figure 1E; control embryos SR = 93.4%±7.54%; 1%EtOH-treated embryos SR = 81.62%±11.65; 2.5%EtOH-treated embryos SR = 44.73%±2.38 *p* = 0.004).

To explore whether transient ethanol exposure resulted in embryos with a range of effects related to those observed during prenatal ethanol exposure, we analyzed the basic features described for FASD. First, embryos showed a reduction in the length of its anteroposterior body axis (Figure 1F). When quantified, the body length of control embryos was of 176.15 mm±8.98 meanwhile embryos exposed to ethanol that survived displayed a shorter body length (1%EtOH-treated embryos 156.80 mm±24.48; 2.5%EtOH-exposed embryos 141.34 mm±37.57). Second, in order to determine the embryonic incidence of pericardial oedema we annotated the number of malformations observed at 72hpf upon transient ethanol-treatment (Figure 1G). Embryos exposed to 1% of ethanol showed an incidence of malformations of 21.99%±15.45 and the rate of oedema increased significantly in the ones exposed to 2.5% ethanol (83.97%±13.91, *p* = 0.001). Finally, when we measured the eye diameter we observed that in control embryos this was 18.04 mm±2.54, and did not vary from embryos exposed to 1% of ethanol (15.65 mm±3.70 m) (Figure 1H). However, embryos exposed to 2.5% of ethanol showed a significant reduction in eye diameter (11.16 mm±3.82; *p* = 0.001). These analyses allowed us to conclude that the best ethanol concentration to assess effects during embryonic development without having too much mortality was 1% ethanol.

In summary, these results indicate that ethanol exposure during the first 24 h of embryogenesis produces developmental malformations in zebrafish embryos suitable with FASD, even at low ethanol concentration doses (1%). These malformations include: i) reduction of body length, ii) high incidence of malformations, and iii) a reduction of eye diameter. Similarly, other studies have reported comparable effects on zebrafish morphology using a wide range of treatment times and dosages, from 0.5% to 10% ethanol using both binge and chronic treatments [13], [20], [22]–[26]. Interestingly, these phenotypic features are partially overlapping to the effects observed in children with ARBD [5], [27], [28].

### The number of differentiated neurons decreases upon ethanol exposure during embryogenesis

**Figure 2:**
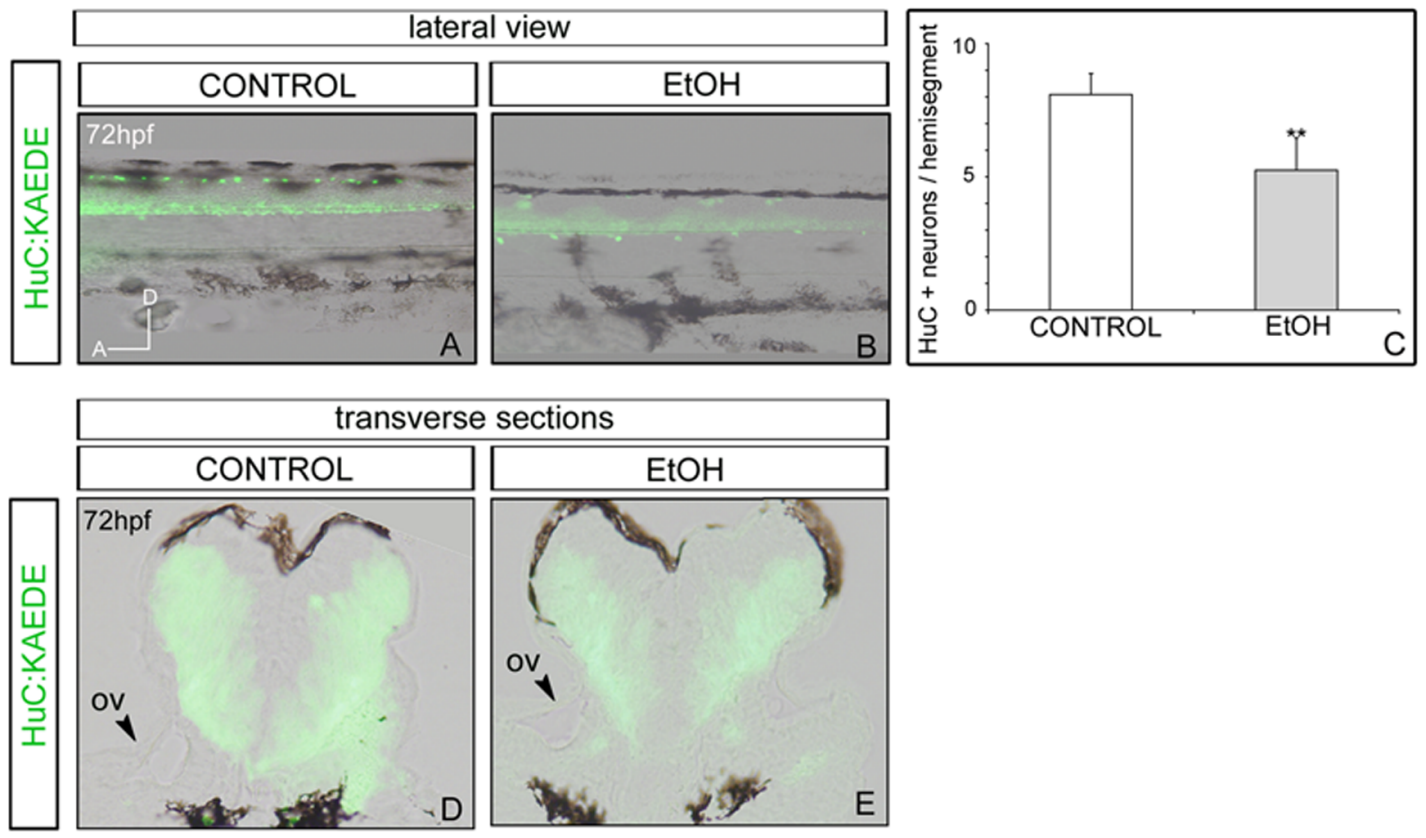
Analysis of Tg[HuC:KAEDE] embryos after ethanol exposure. Control (A,D), or ethanol-treated embryos (B,E) were analysed for neuronal differentiation. (C) Quantification of KAEDE-positive differentiated neurons per hemisegments. Note that EtOH-treated embryos have less differentiated neurons in the spinal cord. (A-B) Lateral views of 72hpf embryos with anterior to the left. (D-E) Transverse sections at the level of the hindbrain. ov; otic vesicle. ** p<0.005 vs. control group.

Next, we wanted to assess the effects of ethanol in neuronal differentiation. We first sought the effect of ethanol exposure in the overall number of differentiated neurons. For this purpose, we employed the stable transgenic fish line Tg[HuC:KAEDE], which provides one of the earliest markers of differentiated neurons in the central and peripheral nervous systems [29], [30]. Embryos were transiently exposed to 1% ethanol and differentiated neurons were analyzed at 72hpf (Figure 2). All embryos displayed the classical pattern of differentiated neurons located along the spinal cord (Figure 2A-B). However, embryos exposed to ethanol exhibited a decrease in the number of differentiated neurons, with a significant reduction in the spinal cord (8.10±0.80 vs. 5.25±1.20; *p* = 0.002; Figure 2C). To rule out the possibility that this decreased number was due to an overall delay in embryonic development, we let treated embryos to develop for longer times and same effects were observed (data not shown). This decrease in the differentiated neurons was observed as well in more anterior regions of the neural tube, such as the hindbrain (Figure 2D-E). Transverse sections at the level of the otic vesicle of control and ethanol-treated embryos showed a high expression of KAEDE in the non-proliferative zone all along the neural tube; however, the KAEDE-positive domain in embryos exposed to ethanol is smaller than in control ones (Figure 2D-E). Overall, these results suggest that the early effects of ethanol are mainly causing a decrease in the number of differentiated neurons along the neural tube. In accordance with our results, some reports showed that ethanol alters *Sox2*, *Oct4* and *Nanog* gene expression program related to neuronal differentiation in differentiating human neural stem cells [31]–[34].

### Differential effects of ethanol exposure on distinct neuronal subpopulations

**Figure 3:**
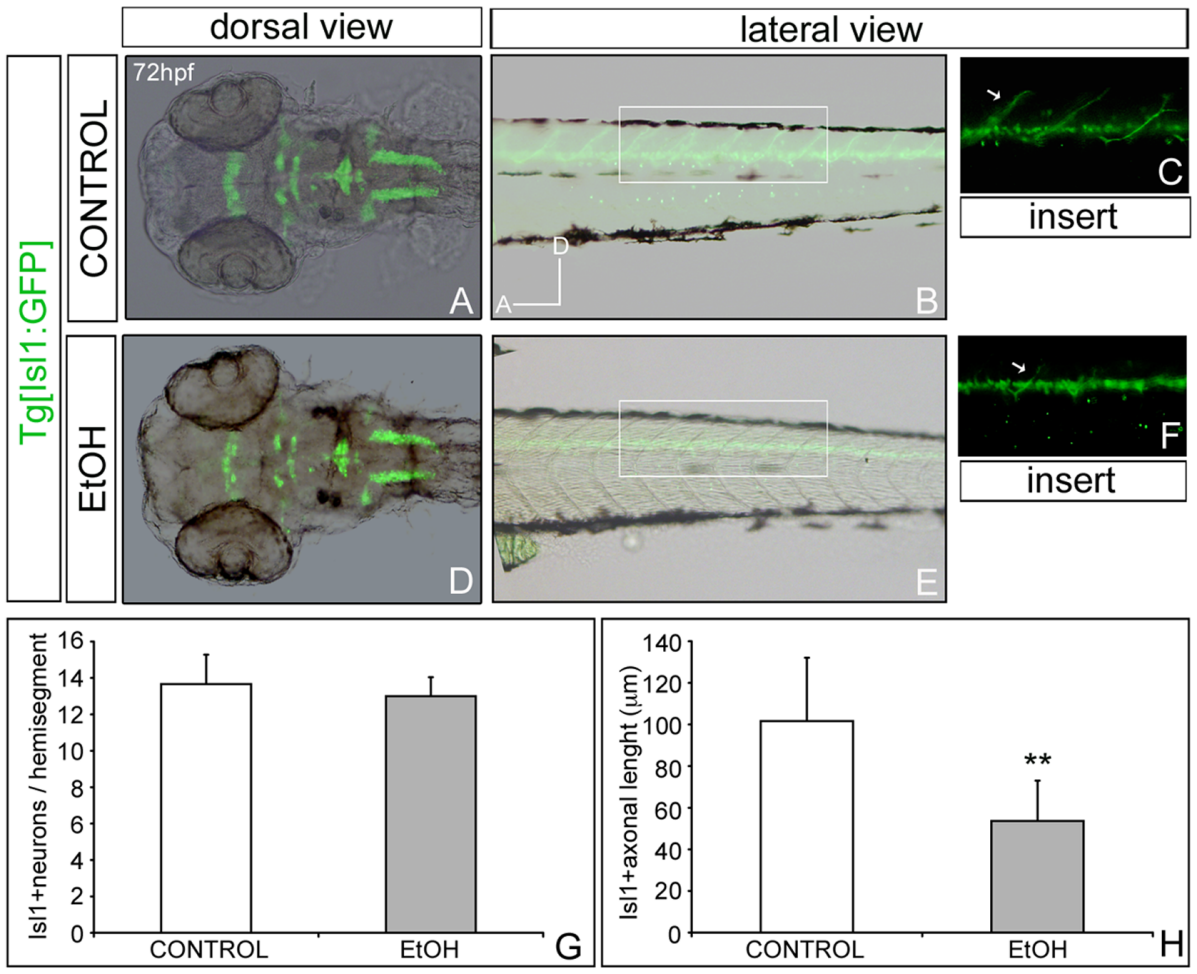
Effects of ethanol in the population of motoneurons. Analysis of Tg[Isl1:GFP] non-exposed embryos (A-C) and ethanol-exposed (D-F). (G) Quantification of GFP-positive motoneurons in specific hemisegments and (H) measurement of axonal lenght. Note that there is not an overall change in the pattern of motoneurons or number of them in treated embryos, although axonal length is diminished.

**Figure 4:**
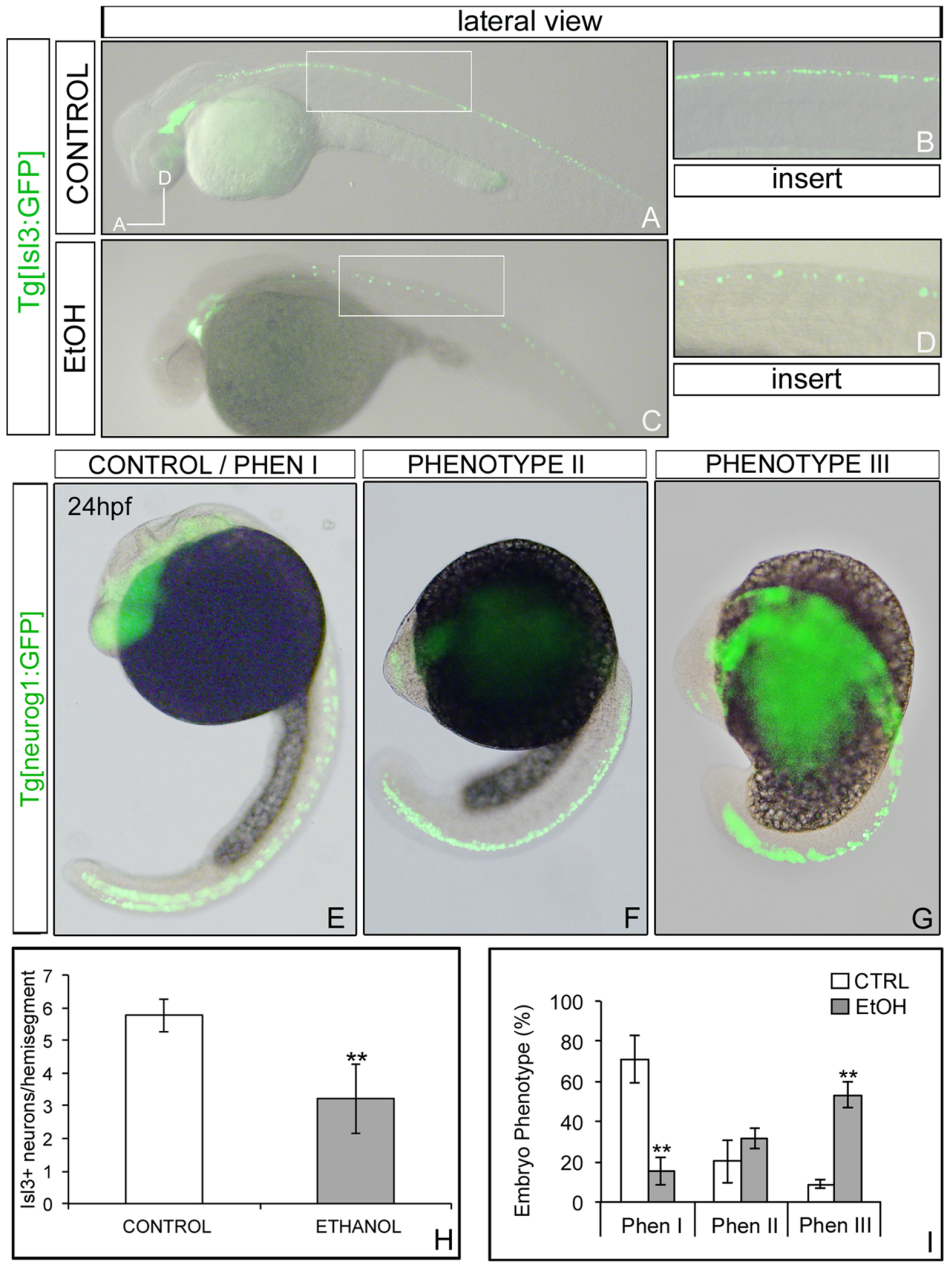
Effects of ethanol in specific neuronal populations. Analysis of Tg[Isl3:GFP] non-exposed embryos (A-B) and exposed to ethanol (C-D) expressing GFP in the primary sensory neurons. (E-G) Tg[neurog1:GFP] embryos treated with ethanol display a graded phenotype: (E) Phenotype I embryos showed a normal development with high levels of GFP along the CNS, (F) Phenotype II embryos showed a delay in their development but displayed normal levels of GFP, and (G) Phenotype III, which has a very short body axis and GFP levels not grossly affected. (H) Quantification of GFP-positive sensory neurons in specific hemisegments. Note the decrease in the number of Isl3:GFP neurons per hemisegment in the ethanol-treated embryos. (I) Quantification of the penetrance of the phenotype in Tg[neurog1:GFP] embryos. ** p<0.005 vs. control group.

In order to identify which subpopulation of differentiated neurons was affected by early ethanol exposure, we used transgenic fish lines expressing GFP in specific neuronal populations (Figures 3 and 4). First, we analyzed the effects on motoneurons by the use of the Tg[Isl1:GFP] line (Figure 3). Isl1 has been identified as the earliest marker for developing motoneurons and its expression is initiated soon after the final mitotic division [35]. At 72hpf, the non-treated embryos showed expression of GFP in all the cranial motoneurons and primary motoneurons located in the ventral region of the spinal cord (Figure 3A-B). When embryos exposed to ethanol were analyzed, neither gross defects in the position, pattern and morphology of cranial or spinal motoneurons were observed (Figure 3D-E), or in the number of motoneurons per hemisegment (Figure 3G). However, axons innervating the dorsal musculature were shorter when compared with the control (Figure 3C,F) and when the axonal length was measured we observed a consistent reduction in the longitude of the main dorsal axon in ethanol-treated embryos compared with controls (101.63µm±30.51 in control embryos, 53.59µm±19.41 in treated embryos *p*<0.0001; Figure 3H). Thus, ethanol-exposed embryos showed a decrease of 47.26% of the length of the motoneuron axons, although no effect in the differentiation of this neuronal population was observed.

These results demonstrate that embryos exposed to ethanol present severe defects in motoneuron axonal branches and correlate with the previous investigations carried out with different ethanol doses [36], [37]. However, several studies suggest different sensitiveness to ethanol of cranial and spinal motoneurons [36], [37], [38]. Using other animal models, some authors have shown that ethanol exposure during gestation causes a significant loss of motoneurons and a reduction of motoneuron diameter [39]. Different windows of exposure and doses might explain the discrepancy between our results and those mentioned before.

To study the effects of ethanol exposure in the development of sensory neurons we used the fish line Tg[Isl3:GFP], which displays GFP in sensory neurons of the spinal cord and in the sensory cranial ganglia [40]. A consistent and robust decrease in the number of cells expressing GFP was observed along the neural tube upon ethanol exposure (Figure 4A-D). When the number of sensory neurons per somite was quantified, a significant decrease was observed in embryos exposed to ethanol when compared with control: the average ratio of sensory neurons per somite in control embryos was 5.8±0.5, versus 3.2±1.1 detected in the ethanol-treated embryos (*p* = 0.001) (Figure 4H). This robust effect in primary sensory neurons upon ethanol exposure was associated with a behavioral phenotype: embryos exhibited fewer bouts of swimming after touching the trunk (data not shown). Interestingly, this behavior can be extrapolated to the effect observed in children exposed prenatally to alcohol that display delays in motor skills and reflex development [18], [27], [41], [42].

The next step was to identify whether this decrease in the differentiated sensory neurons was due to a direct effect on the expression of genes associated with neurogenesis. To seek whether ethanol was affecting sensory neuron development due a reduction of the undifferentiated neuronal precursors we took advantage of the zebrafish line Tg[neurog1:GFP], which expresses GFP in all neuronal progenitors. Neurogenin1 (*neurog1*), is a transcription factor expressed in the proliferating neuronal progenitor cells responsible for neuronal specification [43]. After ethanol exposure, we observed a gradient of phenotypes but embryos presented normal levels of GFP expression along the CNS (Figure 4E-G). The penetrance of the phenotype allowed us to subdivide the embryos in 3 main groups according to the strength of the effect (Figure 4I): i) control or phenotype I, which was significantly present in the non-treated embryos (71.0%±11.6) and in a small population of ethanol-treated embryos (15.56%±7.07; *p* = 0.004) (Figure 4E), these embryos develop normally; ii) mild or phenotype II was displayed similarly by embryos exposed to ethanol (31.9%±5.3) and in control embryos (20.1%±10.5) (Figure 4F), this group was partially delayed in their development; and iii) strong or phenotype III was displayed mainly by the ethanol-treated group (Figure 4G; ethanol: 53.21%±6.28, control: 8.89%±1.97; *p* = 0.004). This last group of embryos was curved along the AP axis and took much longer to develop until this stage. These Tg[neurog1:GFP] embryos seem to display a more severe phenotype than the other transgenic embryos. We think that this is due to the fact that these embryos were observed at 24hpf, much earlier than for the other analyses, and most probably this severely affected group constitutes the pool of embryos that do not proceed until 72hpf. Indeed, when we let them to develop, most of them die. However, we cannot exclude this can be due to an effect of the genetic background.

Overall, these results suggest that although ethanol treatment delays embryonic growth, this does not result in gross alterations in the number of neuronal progenitors during early neurogenesis. Some authors have observed alterations in the pattern of gene expression related with neurogenesis and neuronal differentiation using stem cell populations [31], [44], [45]. However, all these studies have been performed using cell cultures, with all the caveats that this implies. Our results suggest that the target of the effects of ethanol exposure during this period is related with changes in the expression of genes related with cell differentiation.

### Effects of ethanol on cell proliferation/apoptosis

**Figure 5:**
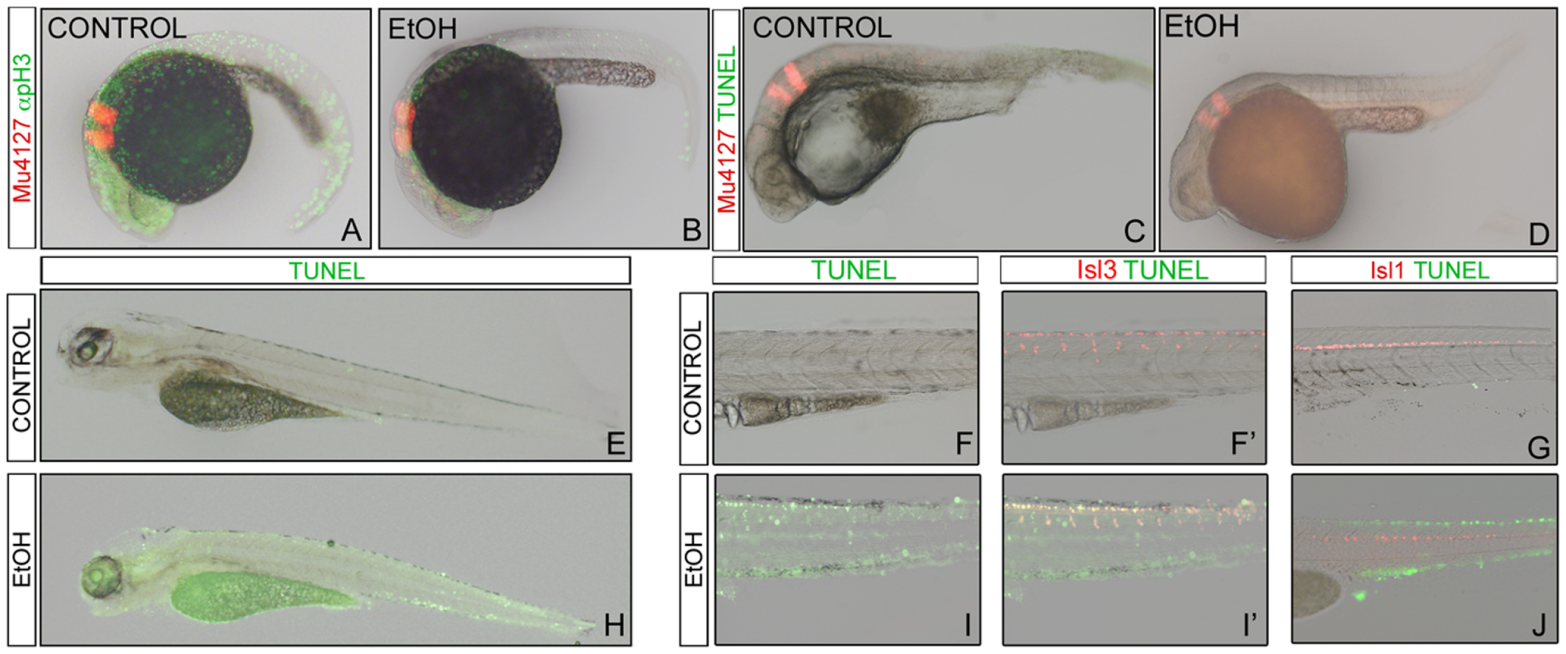
Analysis of cell proliferation and apoptotic cells in control and ethanol-treated embryos. (A-B) anti-pH3 immunofluorescence at 24hpf in order to analyse cell proliferation. (C-J) Cell death visualization using TUNEL assay in 36hpf embryos (C-D), and 5dpf larvae (E-J). Tg[Mü4127:mCherry] embryos were use for landmarks of r3 and r5 in red. (E,H) Apoptotic activity was visualized in whole-mount embryos at 5dpf. Note that ethanol-treated embryos have more apoptotic figures. (F-I) TUNEL analysis (green) in 5dpf embryos displaying red in the primary sensory neurons (F-F′, I-I′) or in motoneurons (G,J). All are lateral views with anterior to the left.

Since neuronal specification was not affected, we hypothesized that loss of differentiated neurons was mainly due to problems in cell proliferation and/or cell death. Thus, we looked for cells undergoing mitosis by anti-pH3 immunostaining. Control embryos showed high levels of pH3-positive cells regularly distributed along the neural tube at 24hpf. On the other hand, pH3-positive cells in the embryos exposed to ethanol were clearly diminished (Figure 5A-B), supporting the idea that ethanol has a strong effect in cell proliferation. To determine whether the reduction in the number of differentiated neurons in the alcohol-exposed embryos was as well contributed by apoptosis, we performed TUNEL experiments (Figure 5C-J). TUNEL-positive cells were few and sparse at 24hpf, both in control and treated embryos (Figure 5C-D). However, when TUNEL analysis was performed at larva stages, the ones exposed to ethanol displayed higher levels of apoptotic events in the whole embryo, including the CNS (Figure 5H) than control larvae. In order to identify which neuronal population was undergoing apoptosis, we made use of Tg[Isl3:GFP] and Tg[Isl1:GFP] embryos (Figure 5F-J). Ethanol-exposed Tg[Isl3:GFP] embryos displayed many more sensory neurons (see red cells, Figure 5I′) undergoing apoptosis (see green cells, Figure 5I-I′) than control embryos (Figure 5F-F′). As expected, Tg[Isl1:GFP] embryos did not show many TUNEL-positive cells along the spinal cord and in the adjacent tissues (Figure 5G), and the few observed apoptotic events did not affect motoneurons (Figure 5G-J). This supports our previous observation that the effects of ethanol in the motoneuron population are very mild.

During embryogenesis, proliferation and differentiation of neural progenitor cells need to be tightly coordinated. In this study, we have demonstrated that transient ethanol exposure results in reduced neuronal cell differentiation due to a breakage in the cell proliferation/apoptosis balance. This may explain why ethanol exposure did not affect the initiation of apoptosis during the first stages of differentiation, but at later stages, the neuronal progenitors failed differentiation and apoptosis events were activated. The effects of alcohol exposure on fetal growth are well known, and there is evidence that ethanol suppresses cell division which may cause retardation in growth [46]. Recently, similar results have been observed using zebrafish [47]. On the other side, ethanol exposure causes extensive cell death when treated from 0 to 24 hpf [15], [16], [48], [49] however the mechanisms involved are unclear. Previous studies revealed a reduction in Sonic hedgehog (Shh) signaling as the major target of ethanol during embryonic development [48], [50], [51]. It is well known that differentiated neuron generation depends on Shh/BMP gradients [52], [53]. These data, along with our observations can be considered potential molecular mechanisms involved in the pathogenesis of FASD.

### Ethanol exposure does not alter hindbrain patterning

**Figure 6:**
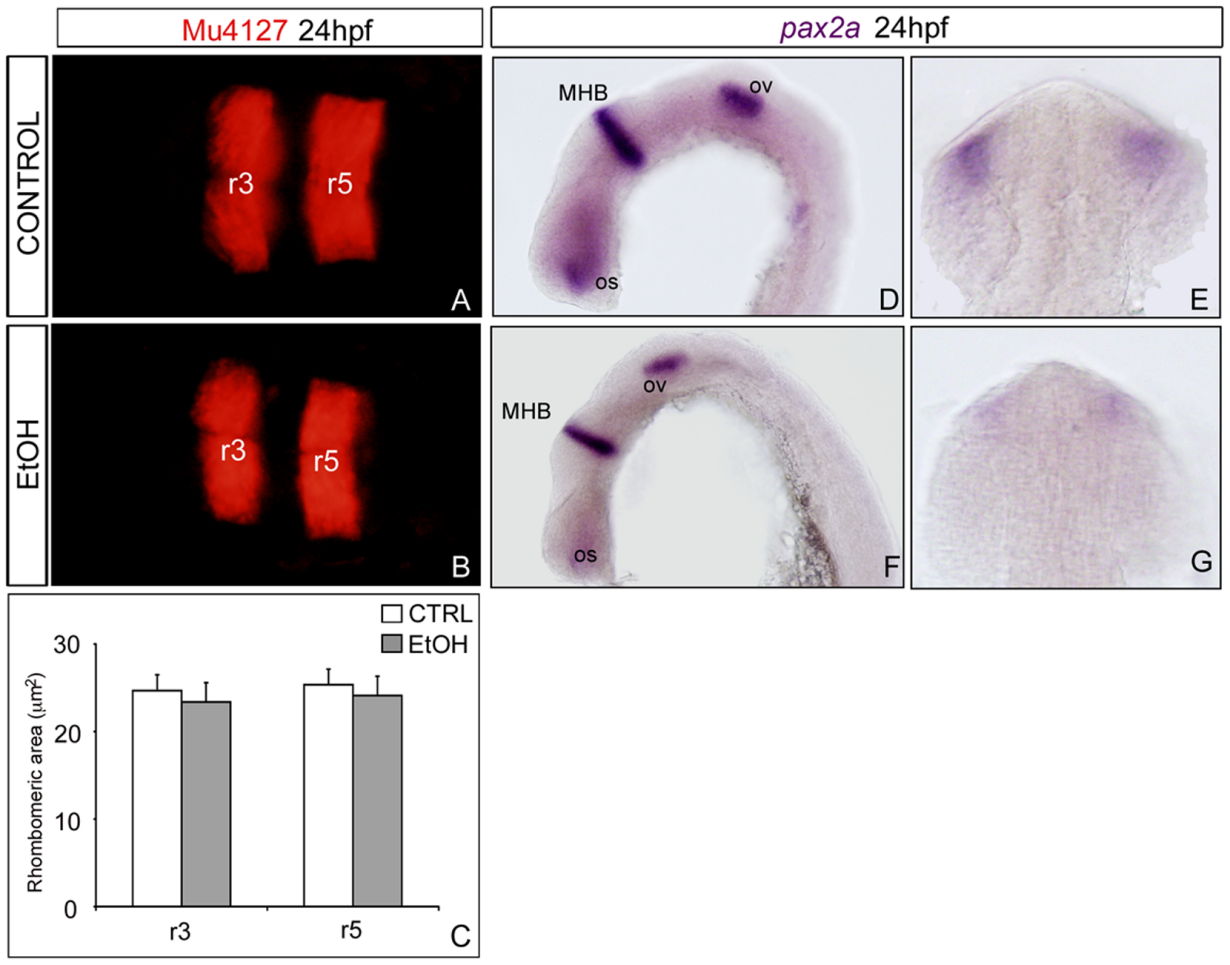
Transient ethanol exposure does not alter either hindbrain patterning or MHB formation. (A,D,E) Control and (B,F,G) ethanol-exposed embryos display no differences in expression of mCherry in r3 and r5 at 36hpf. (C) Quantification of r3 and r5 area, showing no differences upon ethanol exposure. (D-G) In situ hybridization of *pax2a* in 24hpf embryos. (D-E) Control and (F-G) ethanol-exposed embryos showed no differences in the expression of *pax2a* in the MHB and otic placode, however in the ethanol-treated embryos *pax2a* was downregulated in the optic stalks. Mid-Hindbrain Boundary (MHB); optic stalk (os); otic vesicle (ov).

To study the overall effects of ethanol exposure on early CNS development and patterning, we checked the expression of *krx20*, an important transcription factor for the development of r3 and r5 [54]. For this purpose, we used the transgenic zebrafish line named Mü4127 that carries mCherry in the *krx20* 3′UTR, therefore driving expression of the fluorescent protein mCherry to r3 and r5 [55], [56]. As shown in Figure 6A-B, embryos exposed to ethanol did not show any difference compared with control embryos. When the surface or r3 and r5 were measured, no reduction in r3 (23910.70µm^2^±1540.73 vs. 22289.50µm^2^±3113.48) and r5 (24468.00µm^2^±1108.97 vs. 21395.10µm^2^±2276.95) territories was observed (Figure 6C). These results demonstrate that acute ethanol treatment during early developmental stages does not affect the integrity of the hindbrain.

Secondly, we evaluated any possible effects in the Mid-Hindbrain Boundary (MHB) formation by *pax2a* expression. *pax2a* is expressed, in addition to the MHB, in other regions of the embryo such as the optic stalk or the otic placode and vesicle (Figure 6D) [35], [36]. No alterations in MHB formation were induced by 1% ethanol exposure during embryogenesis (Figure 6D-E), nor in the otic placode. On the other hand, a clear reduction in the expression of *pax2a* was detected in the region of the optic stalk of the embryos exposed to ethanol (Figure 6E,G) as recently reported in Zhang C *et al.* [57].

Similarly to neurogenesis, early patterning of the CNS was not affected by early exposure to ethanol. Although there are not big changes in the overall hindbrain patterning, the FASD-like craniofacial malformations may probably be due to apoptosis of neural crest cells (NCC) streams deriving from the hindbrain [7], [58], [59]. In zebrafish, platelet-derived growth factor receptor alpha (Pdgfra) regulates the appropriate migration of NCC that will generate the midface skeleton [60]. Correct Pdgfra signaling involves PI3K activation and increasing PI3K function in ethanol-treated pdgfra mutant embryos rescues both survival and migration in NCC [59]. Although *pax2a* expression is not affected in the MHB, we observed a decrease in the expression of this gene in the region of the optic stalk after the exposure of ethanol, as previously reported using other tools [38]. These results and other studies that have used different gene expression analyses and experimental design [14], [26], [48], [57] support the idea that eye development is a major target in the development of the disease.

## Conclusions

In this work, we studied the effects of transient ethanol exposure during early stages of embryonic development taking advantage of stable and well-characterized transgenic zebrafish lines, which express fluorescent reporter proteins in specific neural territories and subsets of neurons. We demonstrate the use of zebrafish as an emerging attractive model for fast and efficient studies for ethanol teratogenic effects since offers the possibility of imaging organ formation in whole embryos. This is a powerful strategy to study cellular processes that often cannot be replicated *in vitro* [61].

Further research will be needed to clarify the relationship of these impairments observed in the zebrafish model with the effects observed in children with FASD. Furthermore, the duration and developmental timing of exposure, as well as the ethanol concentration employed, will need to be considered. Recently, Flentke *et al.* [49] suggested that even binge ethanol exposures higher than 2% (*v/v*) were of limited clinical relevance. Finally, it needs to be determined which factors are contributing to the impairment in the children with FASD, especially in the motor area, to plan an appropriate treatment.

## Materials and Methods

### Animals

Zebrafish embryos (*Danio rerio*) were produced by paired mating of adult fish in the Parc de Recerca Biomèdica de Barcelona (PRBB) zebrafish facility by standard methods. All lines were maintained individually as inbred lines. Mü4127 expresses mCherry specifically in rhombomere (r)3 and r5 [19]. Tg[neurog1:GFP] is a marker of neuronal specification [62]. Tg[Isl1:GFP] expresses GFP on forebrain nuclei and it is a marker of motoneurons [63]. In Tg[Isl3:GFP] (also called isl2b) GFP is expressed in the afferent sensory neurons [40]. Tg[HuC:KAEDE] line provides one of the earliest markers of differentiated neurons in the central and peripheral nervous systems [29]. All procedures used have been approved by the institutional animal care and use ethic committee (Parc de Recerca Biomèdica de Barcelona Institutional Animal Care and Use Committee), and implemented according to national rules and European regulations.

### Ethanol treatments

A titration of ethanol concentrations was performed and two concentrations of exposure were chosen: 1% and 2.5% (VWR, Radnor, USA) [19], [20], [22]. All solutions were prepared by dilution of absolute ethanol in system water. When embryos reached the desired developmental stage, they were gently transferred into 6-well microtitre plates (Costar 3599, Corning Inc., NY) at a density of 50 embryos per well. To minimize handling stress, embryos were not dechorionated. We compared the effects of the presence of the chorion in ethanol-related phenotypes and no differences were observed (data not shown) in agreement with another study that evaluated this effect [19]. Treatments were performed from 10hpf to 24hpf. At this stage, solutions were changed with system water and embryos were taken for analysis or allowed to grow until the desired stage.

### In situ hybridization and immunolabeling

Whole-mount in situ hybridization was performed as described previously [64]. Digoxygenin-labeled riboprobes were transcribed from cDNAs encoding *pax6a* (Zecca et al, under review) and were detected with NBT/BCIP substrate. For immunostaining, embryos were fixed in 4% paraformaldehyde (PFA) overnight (O/N) at 4°C and washed in Phosphate Buffered Solution with 0.5% TritonX100 (PBS-T). Embryos were generally dehydrated in 100% MetOH at −20°C O/N and permeabilized with Proteinase K (Invitrogen) at 10 µg/ml (for 24–48hpf embryos) or 25 µg/ml (for 48hpf embryos and older) at room temperature (RT) for 10–15 min. Afterwards, embryos were incubated O/N at 4°C with pAb anti-GFP [1∶400] (Torrey Pines, La Jolla, CA), pAb anti-DsRed [1∶300] (ClonTech, Palo Alto, CA) or phospho-histone3 (pH3) [1∶500] (Upstate Biotech, Lake District, NY) in blocking solution. Secondary antibodies conjugated with Alexa Fluor488 (green) or Alexa Fluor568 (red) [1∶800] (Invitrogen, Carlsbad, CA) were used. Whole-mount embryos were imaged under a Leica DM6000B fluorescence microscope.

### Cryostat sectioning

Embryos were fixed in 4% PFA, cryoprotected in 15% sucrose, and embedded in 7.5% gelatine/15% sucrose. Blocks were frozen in 2-methylbutane (Sigma) to improve tissue preservation, and then 20µm sections were done on a LeicaCM1510-1 cryostat.

### TUNEL assay

Apoptotic cells were detected by terminal deoxynucleotidyl transferase-mediated dUTP nick end-labeling (TUNEL) technology using In Situ Cell Death Detection Kit, Fluorescein (Roche Applied Science, Mannheim, Germany). Briefly, whole embryos fixed in 4% PFA and dehydrated in 100% MetOH were permeabilized with Proteinase K at 25 µg/ml. Embryos were preincubated with TUNEL mixture during 60 min according to the manufacturer's instructions. Embryos were washed (3×15 min) in PBS-T and cleared in glycerol: PBS (1∶1 *v/v*).

### Statistical analysis

Statistical analysis was performed using the one-way analysis of variance (ANOVA) or Student's t-test (SPSS for Windows (version 14)). Data for oedema, embryo length and eye diameter, were expressed as mean ± SD. In embryos treated with ethanol, GFP-expressing spinal motor or sensory neurons in the trunk region were counted per specific hemisegments following a procedure used earlier, which is based in the use of the three hemisegments following the distal end of the yolk extension [65]. The values from 10 embryos each per experimental group were averaged to obtain the number of neurons/hemisegment. Relative motor axon (GFP-positive) lengths were measured using a micrometer. Differences were considered as statistically significant when p<0.005.
